# Health-care organization for the management and surveillance of SARS-CoV-2 infection in children during pandemic in Campania region, Italy

**DOI:** 10.1186/s13052-020-00928-y

**Published:** 2020-11-16

**Authors:** Francesco Nunziata, Eugenia Bruzzese, Marco Poeta, Luca Pierri, Andrea Catzola, Gian Paolo Ciccarelli, Edoardo Vassallo, Emma Montella, Andrea Lo Vecchio, Alfredo Guarino

**Affiliations:** 1 Department of Translational Medical Sciences – Paediatrics Section, University of Naples Federico II, via Pansini 5, 80134 Naples, Italy; 2 Department of Public Health, University of Naples Federico II, Naples, Italy

**Keywords:** Children, Severe acute respiratory syndrome coronavirus 2 (SARS-CoV-2), COVID-19, Management

## Abstract

**Background:**

In comparison with adults, severe acute respiratory syndrome coronavirus (SARS-CoV-2) infection in children has a milder course. The management of children with suspected or confirmed coronavirus disease (COVID-19) needs to be appropriately targeted.

**Methods:**

We designed a hub-and-spoke system to provide healthcare indications based on the use of telemedicine and stringent admission criteria, coordinate local stakeholders and disseminate information.

**Result:**

Between March 24th and September 24th 2020, the Hub Centre managed a total of 208 children (52% males, median age, 5*.*2, IQR 2–9.6 years) with suspected or confirmed COVID-19. Among them, 174 were managed in cooperation with family pediatricians and 34 with hospital-based physicians. One hundred-four (50%) received a final diagnosis of SARS-CoV-2 infection. Application of stringent criteria for hospital admission based on clinical conditions, risk factors and respect of biocontainment measures, allowed to manage the majority of cases (74, 71.1%) through telemedicine. Thirty children (28%) were hospitalized (median length 10 days, IQR 5–19 days), mainly due to the presence of persistent fever, mild respiratory distress or co-infection occurring in infant or children with underlying conditions. However, the reasons for admission slightly changed over time.

**Conclusion:**

An hub-and-spoke system is effective in coordinate territorial health-care structures involved in management paediatric COVID-19 cases through telemedicine and the definition of stringent hospital admission criteria.

The management of children with COVID-19 should be based on clinical conditions, assessed on a case-by-case critical evaluation, as well as on isolation measures, but may vary according to local epidemiological changes.

## Introduction

The novel 2019 coronavirus disease (COVID-19) pandemic has disrupted social and economic life and created a global medical emergency. Due to the dramatic spread of the disease and the lack of rapid, reliable, and widely available diagnostic tests, social distancing, quarantine, and isolation measures have been applied so far [1]. Evidence concerning the risk and severity of COVID-19 in children remains relatively reassuring compared to the situation with adults [2]. Children have similar clinical hallmarks as adults; however, their symptoms are milder and the risks of complications, commonly observed in adults and particularly in elderly persons, are rare. However, relevant clinical studies have been diversely conducted, reflecting the heterogeneity of population enrolment criteria as well as of local management. In Wuhan, China, < 1% of 72,000 patients were found to be aged < 10 years; in USA 8.7% patients with SARS-CoV-2 infection were found between 0 and 17 years [3, 4], (https://covid.cdc.gov/covid-data-tracker/#demographics)*.* At the beginning of pandemic lethal cases reported in USA were 67 (0–14 years)[5] (https://www.cdc.gov/nchs/nvss/vsrr/covid_weekly/index.htm), in Italy 4 cases (0–9 years) [6] (https://www.epicentro.iss.it/coronavirus/bollettino/Bollettino-sorveglianza-integrata-COVID-19_1-settembre-2020.pdf)*.* Severe and critical cases of COVID-19 have been confirmed in patients with coexisting conditions [7]*.* A more severe disease scenario was revealed from a case series in Spain reporting 41 children admitted with COVID-19-related respiratory symptoms and fever. Compared with children with similar clinical symptoms, that is, with suspected COVID-19 but whose test results were confirmed as negative, the general clinical conditions and symptoms were more severe, and a total of 4 children were referred to a paediatric intensive care unit, although no deaths were reported. However, it is difficult to reliably estimate what the risk is of a severe clinical outcome for children with COVID-19 in these different reports due, as noted, to heterogeneous enrolment criteria and disease management [8, 9]. One report summarizes the clinical features of nine infants aged < 1 year with SARS-CoV-2 infection. All had been in contact with at least one affected family member and all eventually recovered. Clearly since no infant can take protective measures and children are a major source of infection, caregivers have to be protected. However, the authors did not report the rate of SARS-CoV-2 infection among the caregivers involved [10]. Another recent study suggest that children are not significant drivers of the COVID-19 pandemic [11].

*A* COVID-19 diagnosis needs to be obtained quickly and isolation applied strictly; however, isolation is not easy to ensure for children admitted to hospital. Even quarantine at home can be highly challenging. Management of children is virtually impossible without the constant presence of a caregiver, particularly for infants and young children who need to stay in one room for at least two weeks (the usual period of COVID-19 positivity) and who are dependent on adults for their basic needs, including feeding and hygiene. Furthermore, two additional factors are somewhat more worrying in relation to children. The first is that a child with few or no symptoms may spread SARS-CoV-2 infection to at-risk contacts, such as grandparents, and with a high probability to the direct caregiver, with highly dangerous consequences for such caregivers. The second is that certain children belonging to at-risk groups, particularly those with chronic conditions or immune suppression, may be at high risk (similar to the situation with adults) and should be closely observed clinically. Considering these factors, we designed a system for improved management of children exposed to or infected with SARS-CoV-2, in cooperation with primary care paediatricians (known as family paediatricians (FPs) in the Italian healthcare system) as well as with emergency care paediatricians. In the Italian public health system, all children are taken care of by FPs, who are responsible for disease prevention and care of all the children in a family. In general, the FPs have a complete knowledge of the entire family setting and particularly of the children, including their clinical history, immunisation record, and recent health status, as well as their social and living conditions.

A COVID-19 specific paediatric reference centre has been established to manage paediatric cases in the Campania region in Italy. This Hub Centre provides information to other healthcare institutions and primary care paediatricians, and coordinates medical services for children aged < 14 years. Here we describe the specific pathway used to manage children with or exposed to COVID 19 infection based on the use of telemedicine and of stringent admission criteria.

## Methods

### Setting and definitions

Campania is the most populous region in southern Italy, comprising approximately six million residents, of whom approximately 500,000 are children. In this setting, a hub-and-spoke system for the management of children aged < 14 years with COVID-19 was set up, with the Hub Centre coordinating all medical services involving children’s admissions to a paediatric COVID-19-specific unit at the Hospital Federico II, Naples. The peak of COVID-19 infection occurred slightly later in the Campania region compared to the massive outbreak in Lombardy, [12] which allowed for more effective preparation. A suspected case was defined as a child with fever and respiratory symptoms AND exposure to COVID-19 through COVID-19-infected relatives or cohabitants or through direct contact with established infection OR as a child living within a designated red area (generally a small village or neighbourhood with fewer than 20,000 persons in which a cluster of SARS-CoV-2 infection had been found) and where extremely strict quarantine measures had been implemented. This definition was applied in cooperation with emergency hospital units and other hospitals. A child was considered for hospitalisation to the specialised unit only if specific criteria in relation to COVID-19 were met and always after a telephone consultation with paediatric infectious diseases specialists to discuss and approve hospital admission.

### Organisation of the management of children with or exposed to COVID-19 infection

A taskforce of 2 senior paediatricians and 6 paediatric residents was set up to work in the specialised unit. Residents in their 4th or 5th year of paediatrics formation were intensively trained at an early stage of the COVID-19 pandemic and enrolled in the taskforce. They updated incoming scientific information, oversaw a call centre, and were on call under the supervision of senior university paediatricians highly skilled in infectious diseases.

The unit consisted of 5 single rooms with strict isolation criteria and a triage room in which children were screened for admission in cases of suspected SARS-CoV-2 infection. Separate rooms for donning, doffing, and disposing of personal protective equipment were available. The call centre, which ran 24 h a day 7 days a week, was made available to family physicians and other physicians to discuss medical assessments and for consultations. The management decision resulting from a call to the call centre could include one of the following options: hospital admission to the specialised unit, COVID-19 triage to directly check the infection status and clinical conditions, quarantine at home, management at home of a suspected case or cases, management of a proven COVID-19 infected case or cases, or watchful waiting and update for re-evaluation within 24 h. The triage process for COVID-19 included clinical evaluation and microbiological examination using a COVID-19 swab test. In all cases, feedback and follow-up measures were offered by the specialised unit to the FP concerned. To establish SARS-CoV-2 criteria for admission, we reviewed the clinical criteria for hospitalisation in children with influenza-like-illness, pneumonia [13, 14], or bronchiolitis and applied those relevant risk factors in determining a severe SARS-CoV-2 infection course and outcome (among children aged < 1 year). These criteria include: severe clinical conditions or urgent need for hospital procedures, signs of respiratory distress, or the presence of underlying severe chronic diseases. In addition, at least in the first phase of pandemics, even in the absence of clinical criteria, biocontainment criteria were applied. A child testing positive for COVID-19 was admitted if he/she was residing with cohabitants at risk due to older age or chronic diseases and because there was no alternative means to ensure home protective isolation, or if a child was in need of care and there were no available persons in that particular setting. Characteristics of the home in relation to COVID-19 management, specifically any options for effective protection of at-risk individuals, were also considered. The relevant criteria (globally defined within the context of hospitalisation for preventing the spread of COVID-19) were discussed on a case-by-case basis by the FP concerned and an expert at the call centre. This approach allowed more effective matching of relevant clinical and infection prevention criteria with the actual patient as well as information to be obtained concerning family composition and daily living conditions, resulting in optimal case management.

## Results

**Fig. 1:**
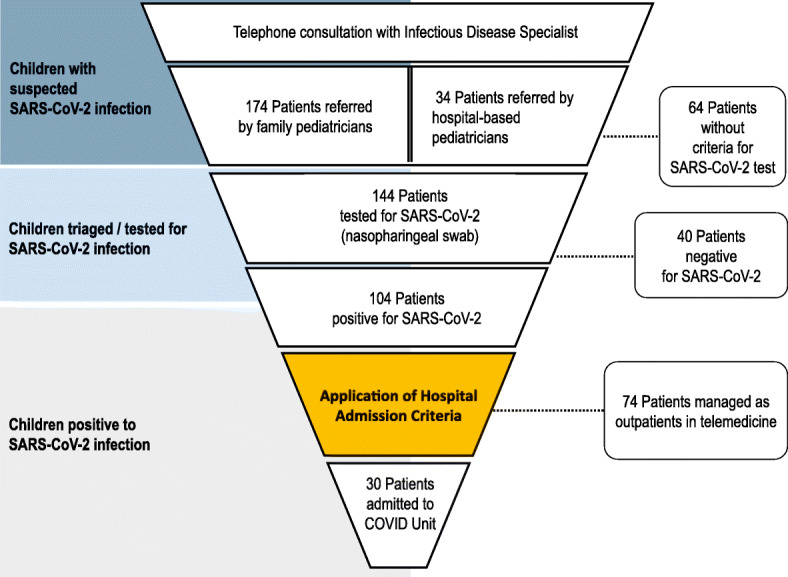
Management pathway of children with COVID-19

A total of 208 children (109 males, 52.4%; median age, 5*.*2; IQR 2–9.6; range 2–15) with suspected COVID-19 were managed by the Hub Centre during the study period (Fig. 1). These included 174 children (median age 5, IQR 1.5–9.6 years) managed in cooperation with FPs and 34 children (median age 3.75; IQR 0.4–12 years) referred from other regional hospitals. The main reasons for contacting the Hub Centre and related decisions taken are reported in (Table 1**)**. One hundred-four patients (50%) received a final diagnosis of SARS-CoV-2 infection through RT-PCR on nasopharyngeal swab (Fig. 1). Those children were managed either through telemedicine or hospitalization according to their clinical conditions, risk factors and respect of biocontainment measures.

**Table 1:** Reasons for telephone consultations and decisions taken

	Total cases ***N*** = 206, (%)	Suspected cases ***N*** = 104, (%)	Confirmed cases ***N*** = 102, (%)
**Type of request**			
General information about management of respiratory symptoms and fever in children with suspected COVID-19	90 (44)	90 (87)	0 (0)
Indications for microbiology examination	60 (29)	57 (55)	3 (3)
Request for admission (general, not referring to clinical cases)	28 (14)	6 (6)	22 (22)
Indications for management of exposed children	104 (50)	104 (100)	0 (0)
Management of COVID-19-positive children living with COVID-19-negative at-risk adults	33 (16)	26 (26)	7 (7)
Request for admission (specific)	28 (14)	6 (6)	22 (22)
Prevention of infection (isolation/quarantine) generally	206 (100)	104 (100)	102 (100)
**Decisions taken following telephone consultations**			
Management of a suspected case	104 (50)	104 (100)	0 (0)
Management of a confirmed case	102 (50)	0 (0)	102 (100)
Watchful waiting	180 (87)	100 (96)	80 (78)
Isolation of a COVID-19-infected child where there was risk to a cohabitant	7 (3)	0 (0)	7 (7)
COVID-19 triage	26 (13)	26 (25)	0 (0)
Admission to the paediatric COVID-19 specialist unit	30 (15)	8 (22)	22 (28)

### Telemedicine

In our study population, most children showed mild-to-moderate clinical features, and the majority of children (74, 71.1%) were managed in telemedicine with FPs and caregivers.

A telephone call lasted 13 min in average and generally led to a shared decision between the experts within the Hub Centre and the paediatricians. At the beginning of the pandemic, most calls reflected a lack of knowledge and tools to manage the resulting issues. A major challenge emerging from the telephone consultations, was to make clear to the paediatricians that stopping the spread of infection was an issue as important to consider as a child’s clinical conditions. In the subsequent phase, the reasons for calling became more specific and centred on diagnostic and clinical issues.

The availability of a follow-up call was reassuring to FPs; however, further contact rarely occurred and was uneventful in most cases.

In certain cases, swabs were obtained, often because of the presence of cohabitants at risk of infection and where isolation at home might be required. However, delay in obtaining swab results (12–36 h) was an operational barrier, mainly at the beginning of pandemic.

Furthermore, the presence of at-risk persons living in the home was an indication to separate a suspected SARS-CoV-2-infected individual from non-infected individuals. Biocontainment, defined as the risk of spreading SARS-CoV-2 infection to at-risk cohabitants in the absence of other isolation/quarantine measures, was included as a specific criterion for hospital admission. In four cases, when appropriate precautions were not realizable at home, children were taken to hospital to be isolated (Table 1).

Another issue was the unknown COVID-19 disease status among the cohabitants, which necessitated a temporary separation of all members in the family or the application of preventive measures, where possible, while awaiting the microbiological results.

### Hospitalization

A total of 30 children were hospitalised (median age 1.15 IQR 0.5–4 years, range, 0.1–15 years) with a median length of hospital stay of 10 days (IQR 5–19 days; range 1–26 days). Specific reasons for hospitalisation are listed in (Table 2). In the majority of cases (14, 46.6%) the main indication to hospital admission was the presence of persistent fever, mild respiratory distress or co-infection occurring in infant or children with underlying conditions (Table 2). Notably, more than half of patients (16, 53.3%) aged below one year and 1 was a 23-days-old neonate, supporting the hypothesis that age was a common driver of hospital admission. None of our cases presented severe respiratory distress or needed oxygen support or ventilation. Four children (13.3%) presented a single episode of complex febrile seizures, in three cases the neurological presentation was the main reason for hospital admission (Table 2), and one infant admitted for fever and mild respiratory distress presented seizures during hospitalization. The latter showed no alteration to computed tomography CT scan, and a long-lasting persistence of the virus (25 days), as shown by serial nasopharyngeal swabs*.* A 9-month-old boy was admitted with a diagnosis of SARS-CoV-2 infection, acute diarrhoea, and the clinical feature of a severe sepsis. During hospitalisation, a blood culture tested positive for extensive beta-lactamase-producing Pseudomonas and the infant received appropriate antibiotic treatment, fluid support, and blood transfusion. No respiratory supportive care was needed. A 15-year-old girl, admitted for cough, showed a ground-glass pneumonia confirmed to CT scan and needed antibiotics and anticoagulant therapy with heparin.

**Table 2:** Main reasons for hospitalization of children with SARS-CoV-2 infection, according to admission criteria

Indications for hospital admission	Admitted children N, (%)
*Absolute*	
Fever < 3 months of age	2 (6.6)
Persistence of high-grade fever (> 38.5°) beyond 5 days	1 (3.3)
Oxygen saturation < 92% OR signs of respiratory distress or tachypnoea	2 (6.6)
• 0–2 months = 60 breaths/min	
• 2–12 months = 50 breaths/min	
• 1–5 years = 40 breaths/min	
• > 5 years = 20 breaths/min	
Seizures or neurological symptoms	3 (10)
Lethargy, alteration in consciousness	0 (0)
Need for parenteral rehydration	1 (3.3)
Surgical condition and/or acute pain (es. renal colic, head trauma)	3 (10)
Congenital cyanotic heart diseases	0 (0)
Myocardial enzymes, coagulation, liver indices, or lactate dehydrogenase alteration	0 (0)
*Relative*	
Aged < 12 months OR pre-existing conditions^a^ AND at least one of the following:	
• Persistent fever for 3–5 days	7 (23.3)
• Oxygen saturation < 94% or mild respiratory distress	2 (6.6)
• Extra-pulmonary complications	1 (3.3)
• Co-infections	2 (6.6)
• Prematurity < 34 weeks or small for Gestational Age (< 2000 g)	1 (3.3)
• Reactivation of underlying chronic condition needed hospital procedures (i.e acidosis)	1 (3.3)
Biocontainment (risk of spreading SARS-CoV-2 infection to at-risk cohabitants in the absence of other isolation/quarantine measures)	4 (13.3)

^a^Pre-existing medical conditions include chronic diseases in which an acute infection may trigger reacutization or rapid clinical impairment: diabetes mellitus, metabolic diseases, adrenal insufficiency, renal insufficiency, hepatic insufficiency, cystic fibrosis, immune disorders and ongoing immunosuppressive therapy

**Fig. 2:**
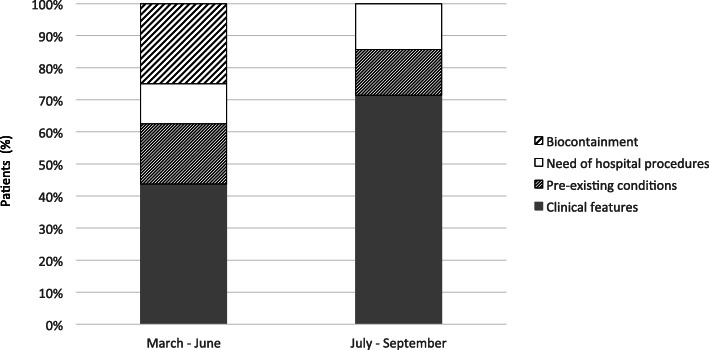
Principal reasons of hospital admission according to pandemic phase

The reasons for hospital admission slightly changed over time (Fig. 2). Although the need of hospital procedures (i.e. intravenous rehydration, diagnostic work-up for trauma of suspected surgical conditions) or the presence of serious underlying conditions were equally distributed, we observed that during the first trimester of activity, about ¼ children was admitted for biocontainment. This criterion was not fulfilled from July 2020, reflecting the increase in the number of cases and a change in local COVID-19 epidemiology.

In many cases, we received by paediatricians a specific request to admit a positive child, because there was little or no knowledge of management options. In such cases, hospitalisation and infection prevention were discussed initially with the FP and subsequently between the FP and the family. Multiple family members were often found to be infected. When the caregiver (often the mother) had to stay in the hospital with the child, discussion occurred on how to organise the family and each member’s role to find the best solution. Related to this, an issue for decision was the infection status of the person taking care of the child within the hospital isolation room, and this issue was openly discussed with the FP and the family before admitting the child. The isolation room was organised as a mini-apartment, with food and hygiene products provided, and the caregiver was responsible for feeding and cleaning the child to limit contact with healthcare workers. In certain cases, SARS-CoV-2 infected parents were cared for by relevant specialists.

## Discussion

Our data showed that a Hub-Spoke health organization is reliable and effective to manage children with SARS-Cov-2 infection, to limit hospitalization to selected cases and reduce inappropriate hospital care.

Since the first phase of epidemics the regional Hub had a close interaction with the epidemiological unit of local health authorities as well as with the primary care pediatricians to identify and monitor positive patients. The role of the Regional Hub should not be limited to the hospitalization of affected patients, but should include the following activities: development of guidelines for local management of children and their relative pathways, coordination of stakeholders (i.e. primary care pediatricians, spoke hospitals and emergency departments and epidemiological unit of local health authorities) and educational activities addressed to practitioners. In addition our clinical data support previous findings that children with COVID-19 do relatively better than adults. However, the risk of SARS-CoV-2 spreading, the unpredictable course of the disease, the fear associated with COVID-19, and the problems associated with feeding and cleaning an infant according to his/her age-related needs while awaiting clearance of viral infection make management of such cases difficult. Most children should be managed at home with little need for direct clinical management. This approach avoids the need for transportation and addresses issues related to wearing protective masks (difficult for a small child) and the risk of a SARS-CoV-2-infected child coming in direct contact with others. Taking a child for a medical visit may be challenging where lockdown conditions are implemented, and most of these infected children can be managed by telephone consultation or through telemedicine. However, in terms of preventing the spread of infection and the application of preventive measures among children, we found that different considerations for children compared to adults need to be considered, which affects the approach to the management of children and of families. Given the lack of in-depth experience in dealing with this crisis, these considerations determined the management strategy and required a case-by-case decision involving the family and the FP in most cases. Among admitted children, only five had a relevant clinical issue, namely, complex seizures, severe general bacterial infection, renal colic, pneumonia, or a real need of medical intervention. All the other children recovered well, only requiring limited supportive measures. Similarly, follow-up at home, performed in collaboration with the FP, was shown to be easy and effective, and there were no major complications among the children. Biocontainment, was identified as a specific indication of hospital admission; in the first phase of pandemic, four children were admitted to the hospital in order to prevent the spread of COVID-19. In most cases, hospitalisation was protracted while awaiting clearance of COVID-19. The need to keep a child in the hospital should be evaluated in relation to options for preventing the spread of infection that could be implemented in the home. The increase in cases, starting from August 2020, did not allow to consider, neither biocontainment as the only admission criterion nor virological recovery as discharge criterion. This study had several limitations, specifically the relatively small sample size and the difficulty of generalising our results, given the heterogeneous organisation of healthcare systems in various settings at national and international level.

## Conclusion

In conclusion, our data indicate that due to the less severe course of SARS-CoV-2 infection in children than in adults the health care system to manage COVID 19 in the paediatric setting may be based on the use of telemedicine and of stringent hospital admission criteria. At the beginning of pandemic every health care system had the need to rapidly set up a specific and sustainable model of care. During the firs two months of pandemic we set up the model, identified barriers and understood the real evolution of the pandemic in children and health-care workers. When the infection was spreading again in the region, as well as nationally, we evaluated the results of the health-care organization and the model we described appeared sustainable and able to avoid inappropriate hospital care.

## Abbreviations

COVID-19: Coronavirus disease
FPs: Family paediatricians
SARS-CoV-2: Severe acute respiratory syndrome
